# The Association of Urine Creatinine With Kidney Stone Prevalence in US Adults: Data From NHANES 2009–2018

**DOI:** 10.3389/fmed.2022.819738

**Published:** 2022-03-29

**Authors:** Xudong Shen, Yan Chen, Yangyang Zhang, Kaiguo Xia, Yang Chen, Zongyao Hao

**Affiliations:** ^1^Department of Urology, The First Affiliated Hospital of Anhui Medical University, Hefei, China; ^2^Institute of Urology, Anhui Medical University, Hefei, China; ^3^Anhui Province Key Laboratory of Genitourinary Diseases, Anhui Medical University, Hefei, China; ^4^Department of General Practice, Wuhu City Second People‘s Hospital, Wuhu, China

**Keywords:** kidney stone, urine creatinine (UCR), national health and nutrition examination survey (NHANES), prevalaence, a cross-sectional survey

## Abstract

**Background:**

The primary objective of this project is to explore the association of urine creatinine (UCR) with the prevalence rate of kidney stones.

**Method:**

The National Health and Nutrition Examination Survey (NHANES) database was employed to conduct a cross-sectional study. The analysis samples included adults aged ≥20 years from five consecutive cycles of the NHANES 2009–2018. The association between UCR and kidney stones was detected using univariate and multivariate logistic regression analyses. Further, subgroup analyses were performed to evaluate the subgroup effects.

**Results:**

After adjustment for all confounders, multiple logistic regression analysis revealed a weak positive relationship between UCR and kidney stone (OR = 1.015, 95% CI: 1.008–1.021). In the subgroup analysis stratified by sex, age, or race, the risk further increased in men (OR = 1.014, 95% CI: 1.005–1.023), women (OR = 1.015, 95% CI: 1.005–1.025), white race (OR = 1.022, 95% CI: 1.013–1.030), aged 40–59 years (OR = 1.017, 95% CI: 1.006–1.028), and aged 60–80 years (OR = 1.017, 95% CI: 1.006–1.028).

**Conclusions:**

Our results confirmed a moderately increased risk of kidney stone formation attributed to high levels of UCR, especially in middle-aged and older adults and the white race. However, because of the cross-sectional design of the study, causal inferences cannot be made.

## Introduction

Kidney stone is a widespread benign urological disorder with overwhelmingly high incidence in the study area (1). A number of latest multicountry survey studies summarized that the prevalence rate of kidney stones in the US (2) was over 10%, whereas in Europe (3) and China (4), it was 9 and 5.8%, respectively. Furthermore, even after formal treatment, a high recurrence risk of kidney stone still prevailed with a relapse rate of 50% within 10 years (4). The development of nephrolithiasis is known to be associated with several predisposing factors, which include male, age, race, high sodium intake, hypercalcemia, monogenic hereditary disorders, obesity, pregnancy, and urine-specific gravity (1, 2, 5–7). Compounded by the increasing overall prevalence of kidney stones, it is alarmingly becoming a serious public social problem and healthcare burden (8). Therefore, to ensure targeted prevention of nephrolithiasis formation and recurrence, the identification of novel modifiable risk factors is the need of the hour.

In fact, kidney stone may lead to intra- or extrarenal urinary outflow obstruction to impair renal function, permanent renal damage, and renal failure which are the most serious complications of renal calculi (9), and creatinine is one of the most common markers in detection and predicting the progression of renal function. The concentrations of UCR can be estimated in both blood and urine. Impaired kidney function affects the serum and urine creatinine (UCR) levels (10). The influence of nephrolithiasis on creatinine is often discussed at present, but what is the impact of creatinine on renal calculi? As is generally known, nephrolithiasis formation in urinary tubules begins with the supersaturation of urine materials (11). The previous researches had centralized mainly on the effects of metabolite changes in urine on kidney stone formation. It also suggests that urine plays a crucial role in the production of kidney stones. As a result, our study could focus only on the effects of UCR on kidney stone formation.

Therefore, to clarify the potential association of UCR with the prevalence rate of kidney stones, we conducted a large population-based cross-sectional study based on the National Health and Nutrition Examination Survey (NHANES) database.

## Materials and Methods

### Study Participants

Data were extracted from the NHANES, which is a nationally representative, cross-sectional survey of the non-institutionalized civilian population designed to obtain nationally representative estimates of the health and nutritional status of the population. Specifically, adult participants were included with recorded data on the history of nephrolithiasis (the responses to the questions ‘‘Have you ever had kidney stones?’’). Survey data were employed in five consecutive cycles from 2009 to 2018, which included demographics, body measures, blood pressure, UCR, standard biochemistry profile, and kidney conditions--urology data. More information about the data is available on the NHANES website.^1^

From 2009 to 2018, a total of 49,693 participants were screened for this study. The exclusion criteria were as follows: (1) incomplete kidney stone survey (*n* = 20,859); (2) unknown kidney stone (*n* = 64); (3) unknown UCR (*n* = 1,757); and (4) unknown serum creatinine (*n* = 1,464); a number of 25,549 subjects aged 20–80 years were included in final analytic cohort.

The study used previously collected public data, and ethical approval was obtained from the institutional review board at the NCHS Ethics Review Board (12).

### Study Variables

Urine creatinine and kidney stones were the most important subjects in this study. The KIQ026 questionnaire data were adopted to determine kidney stones. Urinary creatinine was estimated using the Roche/Hitachi modular P chemistry analyzer, and only in 2013–2014, creatinine was measured on the Roche/Hitachi Cobas 6000 chemistry analyzer. In addition to this, this study considered the following covariates: age, sex, race (Mexican-American, white, black, and other race), education (less than high school, high school or equivalent, college or above, others), marital status (married, unmarried, and others), annual household income, diabetes (yes, no, and other).

In addition to this, this study considered the following covariates: age, sex, race, education, marital status, annual household income, diabetes, systolic blood pressure, diastolic blood pressure, BMI, glycosylated hemoglobin, blood urea nitrogen, total cholesterol, serum calcium, total triglyceride, total protein, serum glucose, serum uric acid, and serum creatinine.

### Statistical Analyses

Data were recorded as categorical and dichotomous or continuous variables. Distributed continuous variables were expressed as mean ± standard deviation; for dichotomous and categorical variables, count proportions were computed. Variations in the clinical characteristics in different groups were determined with the help of chi-square tests (categorical variables) and one-way ANOVA (normal distribution continuous variables) or Kruskal–Wallis H test (skewed distribution continuous variables).

The odds ratio (OR) and 95% confidence interval (CI) were calculated using univariate and multivariate logistic regression models to elucidate the risk of the factors associated with the development of kidney stones. According to the guidelines of the Strengthening the Reporting of Observational Studies in Epidemiology (STROBE) statement (13), the independent association was assessed constructing three logical regression models: (1) unadjusted; (2) adjusted for age, sex, race, education, and marital status; and (3) all of the covariates were adjusted. Furthermore, to identify suitable groups, we conducted subgroups analyses using stratified multivariate logistic regression. Obtaining non-linear relationships between UCR and kidney stone in the subgroups, we performed smooth curve fittings to explore their potential association. Meanwhile, univariate linear regression models and two-piecewise linear regression models were constructed using the same covariates. To identify the best model, a logarithmic ratio test was also done. Furthermore, we applied the model to examine whether a threshold exists. The inflection point connecting the segments based on the model gave maximum likelihood, and it was determined using two steps recursive method. Finally, the association between serum creatinine and UCR was established using smooth curve fittings and two-piecewise linear regression models. Statistical analyses were executed using R version 3.5.3^2^ and the Empower Stats software^3^ with *p*-value < 0.05 as statistically significant.

## Results

After a rigorous screening process, a total of 25,549 participants were considered eligible for the final analysis. Among the eligible individuals, there were 2,412 (9.44%) general population answered a history of kidney stones. The sociodemographic characteristics and descriptive clinical baseline characteristics of all the investigated populations in the two groups are summarized in Table 1. Compared with the non-stone formers, stone formers were found to be elder, men, married, and whites. There also had higher BMI, systolic blood pressure, glycosylated hemoglobin, blood urea nitrogen, total triglyceride, serum glucose, serum uric acid, and serum creatinine levels and had lower serum calcium, total protein, and total cholesterol.

**TABLE 1:** Baseline characteristics of US participants in NHANES from 2009 to 2018.

Characteristic	Stone formers (*n* = 2,412)	Non-stone formers (*n* = 23,137)	*p*-value
Age (years)	55.64 ± 16.25	48.80 ± 17.60	<0.001
Gender			<0.001
Male	1308 (54.23%)	11056 (47.78%)	
Female	1104 (45.77%)	12081 (52.22%)	<0.001
Race			<0.001
White	1545 (64.05%)	11298 (48.83%)	
Black	305 (12.65%)	5016 (21.68%)	
Mexican American	319 (13.23%)	3486 (15.07%)	
Other	243 (10.07%)	3337 (14.42%)	
Education			0.712
Less than high school	576 (23.88%)	5383 (23.27%)	
High school or equivalent	527 (21.85%)	5169 (22.34%)	
College or above	1308 (54.23%)	12562 (54.29%)	
Other	1 (0.04%)	23 (0.10%)	
Marital status			<0.001
Married	1533 (63.56%)	13664 (59.06%)	
Unmarried	877 (36.36%)	9460 (40.89%)	
Other	2 (0.08%)	13 (0.06%)	
Annual Household income (USD)			<0.001
0–19999	317 (13.14%)	2819 (12.18%)	
2000–34999	638 (26.45%)	5619 (24.29%)	
35000–74999	675 (27.99%)	6237 (26.96%)	
>75000	567 (23.51%)	5897 (25.49%)	
Other	215 (8.91%)	2565 (11.09%)	
Diabetes			<0.001
Yes	548 (22.72%)	2823 (12.20%)	
No	1775 (73.59%)	19746 (85.34%)	
Other	89 (3.69%)	568 (2.45%)	
SBP (mmHg)			<0.001
<90	7 (0.29%)	88 (0.38%)	
≥90, <140	1730 (71.72%)	17351 (74.99%)	
≥140	503 (20.85%)	3853 (16.65%)	
Other	172 (7.13%)	1845 (7.97%)	
DBP (mmHg)			0.487
<60	351 (14.55%)	3292 (14.23%)	
≥60, <90	1746 (72.39%)	16692 (72.14%)	
≥90	143 (5.93%)	1308 (5.65%)	
Other	172 (7.13%)	1845 (7.97%)	
BMI (kg/m2)			<0.001
Normal (<25.0)	463 (19.20%)	6734 (29.10%)	
Overweight (25.0–29.9)	793 (32.88%)	7471 (32.29%)	
Obese (≥ 30)	1128 (46.77%)	8720 (37.69%)	
Other	28 (1.16%)	212 (0.92%)	
HBA1C (%)	6.02 ± 1.23	5.77 ± 1.08	<0.001
Blood urea nitrogen (mmol/L)	5.43 ± 2.50	4.90 ± 2.09	<0.001
Total cholesterol (mmol/L)	4.90 ± 1.09	4.96 ± 1.08	0.027
Serum calcium (mmol/L)	2.34 ± 0.10	2.35 ± 0.09	0.022
Total triglyceride (mmol/L)	1.98 ± 2.89	1.75 ± 2.27	<0.001
Total protein (g/L)	70.84 ± 4.58	71.58 ± 4.69	<0.001
Serum glucose (mmol/L)	6.15 ± 2.51	5.70 ± 2.19	<0.001
Serum uric acid (μmol/L)	333.68 ± 90.44	322.62 ± 85.97	<0.001
Serum creatinine(μmol/L)	83.40 ± 38.56	78.57 ± 33.47	<0.001
Urine creatinine(mmol/L)	11.10 ± 6.74	10.93 ± 7.25	0.006

*Statistically significant: p < 0.05; BMI, body mass index; HBA1C, glycosylated hemoglobin.*

### Identify the Risk Factors for Patients With Kidney Stones

To identify the predisposing factors for nephrolithiasis formation, univariate and multivariate logistic regression analyses (the model is adjusted for all of the covariates, except for stratification variable itself) were subsequently constructed, and only those parameters that revealed significant differences in both the two models could be considered effective confounders (Table 2). In our study, confounders were found to be age, gender, race, education, marital status, annual household income, diabetes, BMI, and total triglyceride.

**TABLE 2:** Analysis between confounders and renal stone incidence.

Characteristic	Univariate analysis		Multivariate analysis^a^	
	OR (95% CI)	*p*-value	OR (95% CI)	*p*-value
Age (years)	1.02 (1.02,1.03)	<0.0001	1.02 (1.02, 1.02)	<0.0001
Gender				
Male	Reference		Reference	
Female	0.77 (0.71,0.84)	<0.0001	0.84 (0.76, 0.93)	0.0006
Race				
Mexican American	Reference		Reference	
White	1.49 (1.32,1.70)	<0.0001	1.45 (1.26, 1.66)	<0.0001
Black	0.66 (0.56,0.78)	<0.0001	0.62 (0.52, 0.74)	<0.0001
Other	0.80 (0.67,0.95)	0.0099	0.93 (0.78, 1.12)	0.4703
Education				
Less than high school	Reference		Reference	
High school or equivalent	0.95 (0.84,1.08)	0.4454	1.02 (0.89, 1.16)	0.8059
College or above	0.97 (0.88,1.08)	0.6039	1.13 (1.00, 1.27)	0.0443
Other	0.41 (0.05,3.01)	0.3783	0.45 (0.06, 3.42)	0.4425
Marital status				
Married	Reference		Reference	
Unmarried	0.83 (0.76,0.90)	<0.0001	0.89 (0.81, 0.98)	0.0141
Other	1.37 (0.31,6.08)	0.6778	1.41 (0.30, 6.61)	0.6646
Annual household income (USD)				
0–19999	Reference		Reference	
2000–34999	1.01 (0.88,1.16)	0.8939	0.97 (0.84, 1.12)	0.6655
35000–74999	0.96 (0.84,1.11)	0.5935	0.94 (0.81, 1.09)	0.4003
>75000	0.86 (0.74,0.99)	0.0338	0.83 (0.71, 0.98)	0.0271
Other	0.75 (0.62,0.89)	0.0015	0.76 (0.63, 0.92)	0.0046
Diabetes				
Yes	Reference		Reference	
No	0.46 (0.42, 0.51)	<0.0001	0.63 (0.55, 0.73)	<0.0001
Other	0.81 (0.63, 1.03)	0.0821	0.91 (0.70, 1.17)	0.4490
SBP (mmHg)				
<90	Reference		Reference	
≥90, <140	1.25 (0.58, 2.71)	0.5659	1.06 (0.49, 2.33)	0.8773
≥140	1.64 (0.76, 3.56)	0.2104	1.04 (0.47, 2.29)	0.9260
Other	1.17 (0.53, 2.57)	0.6921	1.05 (0.47, 2.34)	0.9027
DBP (mmHg)				
<60	Reference		Reference	
≥60, <90	0.98 (0.87, 1.11)	0.7558	1.12 (0.98, 1.27)	0.0911
≥90	1.03 (0.84, 1.26)	0.8104	1.21 (0.97, 1.51)	0.0974
Other	0.87 (0.72, 1.06)	0.1685	1.05 (0.47, 2.34)	0.9027
BMI (kg/m2)				
Normal (<25.0)	Reference		Reference	
Overweight (25.0–29.9)	1.54 (1.37, 1.74)	<0.0001	1.32 (1.16, 1.49)	<0.0001
Obese (≥30)	1.88 (1.68, 2.11)	<0.0001	1.62 (1.43, 1.83)	<0.0001
Other	1.92 (1.28, 2.88)	0.0016	1.36 (0.89, 2.06)	0.1524
HBA1C (%)	1.17 (1.14, 1.21)	<0.0001	1.02 (0.96, 1.09)	0.4556
Blood urea nitrogen (mmol/L)	1.10 (1.08, 1.12)	<0.0001	1.01 (0.99, 1.04)	0.4204
Total cholesterol (mmol/L)	0.95 (0.91, 0.98)	0.0065	0.96 (0.92, 1.00)	0.0592
Serum calcium (mmol/L)	0.60 (0.38, 0.95)	0.0309	0.70 (0.43, 1.16)	0.1666
Total triglyceride (mmol/L)	1.03 (1.01, 1.04)	<0.0001	1.02 (1.00, 1.03)	0.0342
Total protein (g/L)	0.97 (0.96, 0.97)	<0.0001	0.99 (0.98, 1.00)	0.1872
Serum glucose (mmol/L)	1.07 (1.06, 1.09)	<0.0001	0.99 (0.97, 1.02)	0.7167
Serum uric acid (μmol/L)	1.001 (1.001,1.002)	<0.0001	1.00 (0.999,1.000)	0.4523
Serum creatinine(μmol/L)	1.003 (1.002,1.004)	<0.0001	1.00 (0.999,1.002)	0.6946
Urine creatinine(mmol/L)	1.003 (1.002,1.009)	<0.0001	1.015 (1.008,1.021)	<0.0001

*^a^model was adjusted by age, sex, race, education, marital status, annual household income, diabetes, systolic blood pressure, diastolic blood pressure, BMI, glycosylated hemoglobin, blood urea nitrogen, total cholesterol, serum calcium, total triglyceride, total protein, serum glucose, serum uric acid, serum creatinine.*

### Association Between Urine Creatinine and Kidney Stones

**FIGURE 1:**
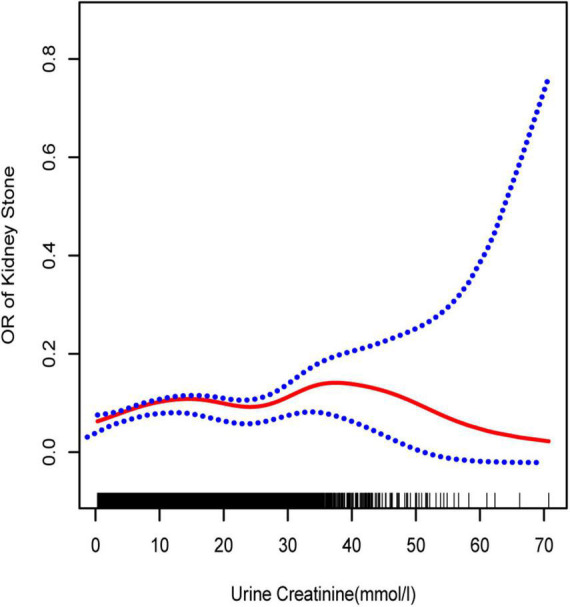
Smooth curve fittings model, adjusted age, sex, race, education, marital status, annual household income, diabetes, systolic blood pressure, diastolic blood pressure, BMI, glycosylated hemoglobin, blood urea nitrogen, total cholesterol, serum calcium, total triglyceride, total protein, serum glucose, serum uric acid, and serum creatinine. The central part of the two blue curves designates 95% CI.

The primary purpose of this study was to clarify the association between UCR and the prevalence of kidney stone formation. Multivariate logistic regression analysis was used. Three models were constructed (Table 3 and Figure 1). After adjusting for all confounding factors fully in model 3, a weak positive trend of UCR with kidney stones formation was evident (OR = 1.015, 95% CI: 1.008–1.021).

**TABLE 3:** Analysis between UCR with kidney stone formation in different subgroups.

	Model 1 OR (95% CI)	Model 2 OR (95% CI)	Model 3 OR (95% CI)
Urine creatinine	1.003 (0.997, 1.009)	1.018 (1.011, 1.024)	1.015 (1.008, 1.021)
Q1	Reference	Reference	Reference
Q2	1.333 (1.179, 1.507)	1.317 (1.163, 1.492)	1.256 (1.107, 1.425)
Q3	1.416 (1.254, 1.599)	1.490 (1.315, 1.688)	1.411 (1.243, 1.602)
Q4	1.205 (1.064, 1.365)	1.508 (1.320, 1.721)	1.419 (1.240, 1.623)
Stratified by sex			
Male	0.994 (0.987,1.002)	1.017 (1.008, 1.025)	1.014 (1.005, 1.023)
Female	1.006 (0.997, 1.015)	1.019 (1.010, 1.029)	1.015 (1.005, 1.025)
Stratified by race			
White	1.020 (1.012, 1.028)	1.025 (1.017, 1.034)	1.022 (1.013, 1.030)
Black	0.988 (0.974, 1.002)	0.999 (0.985, 1.015)	0.997 (0.982, 1.013)
Mexican American	1.002 (0.984, 1.020)	1.013 (0.994, 1.033)	1.015 (0.995, 1.035)
Other	1.007 (0.988, 1.027)	1.016 (0.995, 1.038)	1.008 (0.986, 1.031)
Stratified by age			
20–39	1.000 (0.988,1.011)	1.010 (0.998, 1.023)	1.002 (0.990, 1.014)
40–59	1.013 (1.003, 1.023)	1.021 (1.010, 1.031)	1.017 (1.006, 1.028)
≥60	1.021 (1.012, 1.031)	1.020 (1.009, 1.031)	1.017 (1.006,1.028)

*Model 1 = no covariates were adjusted. Model 2 = model 1 + age, sex, race, education, marital status, were adjusted. Model 3 = model 2 + annual household income, diabetes, systolic blood pressure, diastolic blood pressure, BMI, hemoglobin, blood urea nitrogen, total cholesterol, serum calcium, total triglyceride, total protein, serum glucose, serum uric acid, and serum creatinine were adjusted. The subgroup analysis was stratified by sex, race, or age, not adjusted for the stratification variable itself.*

**FIGURE 2:**
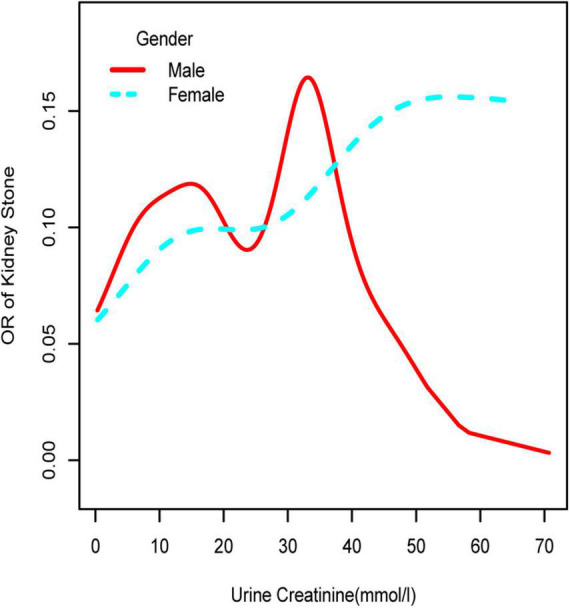
Smooth curve fittings model in the subgroup analysis stratified by gender. Age, race, education, marital status, annual household income, diabetes, systolic blood pressure, diastolic blood pressure, BMI, glycosylated hemoglobin, blood urea nitrogen, total cholesterol, serum calcium, total triglyceride, total protein, serum glucose, serum uric acid, and serum creatinine were adjusted.

**FIGURE 3:**
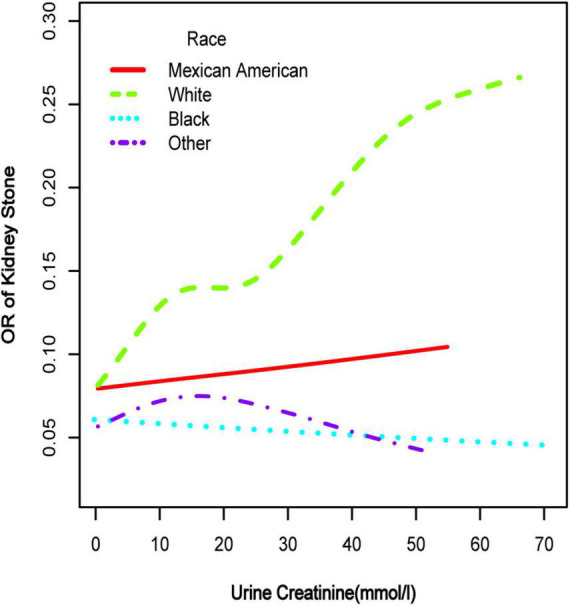
Smooth curve fittings model in the subgroup analysis stratified by race. Age, gender, education, marital status, annual household income, diabetes, systolic blood pressure, diastolic blood pressure, BMI, glycosylated hemoglobin, blood urea nitrogen, total cholesterol, serum calcium, total triglyceride, total protein, serum glucose, serum uric acid, and serum creatinine were adjusted.

**FIGURE 4:**
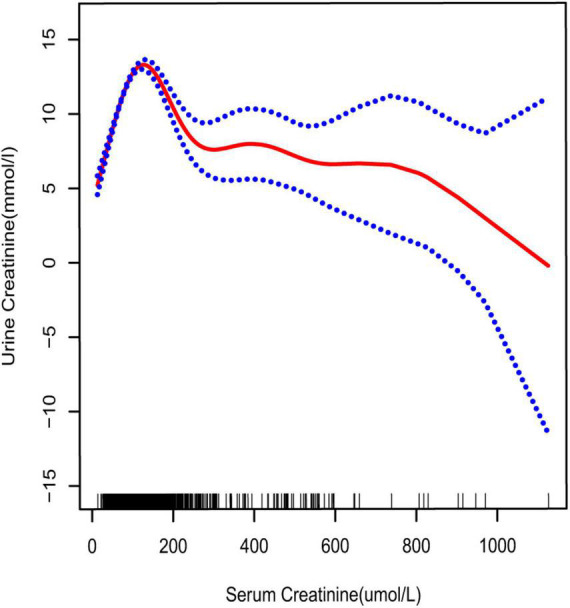
Smooth curve fittings model explained the correlation between serum creatinine and UCR. The central part of the two blue curves represents 95% CI. Each red point denotes a sample. Age, race, education, marital status, annual household income, diabetes, systolic blood pressure, diastolic blood pressure, BMI, glycosylated hemoglobin, blood urea nitrogen, total cholesterol, serum calcium, total triglyceride, total protein, serum glucose, and serum uric acid were adjusted.

Subgroup analyses stratified by UCR quartiles, age, sex, race, and BMI were conducted to address the potential heterogeneous population (Table 3 and Figures 2–4). When compared to Q1, the OR values of the other quartile groups indicated a tendency of progressive growth in the quartile group. Stratified analyses also confirmed similar results. In men (OR = 1.014, 95% CI: 1.005–1.023), women (OR = 1.015, 95% CI: 1.005–1.025), white race (OR = 1.022, 95% CI: 1.013–1.030), aged 40–59 years (OR = 1.017, 95% CI: 1.006–1.028), and aged 60–80 years (OR = 1.017, 95% CI: 1.006–1.028), the risk of kidney stone formation was found to aggravate further with UCR.

**FIGURE 5:**
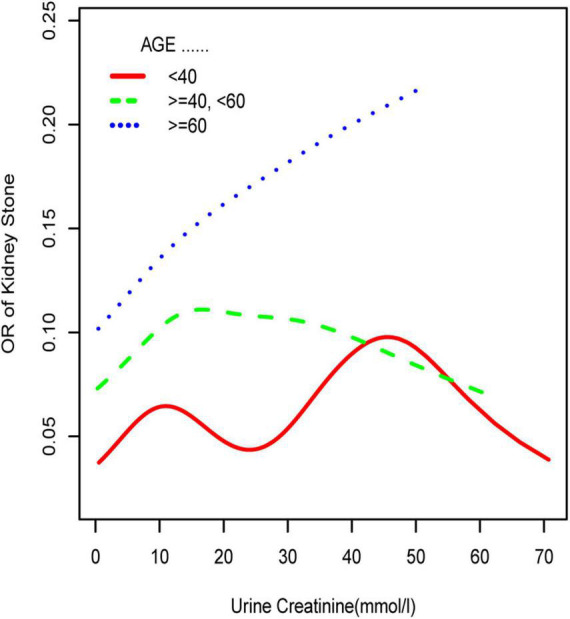
Smooth curve fittings model in the subgroup analysis stratified by age. Sex, race, education, marital status, annual household income, diabetes, systolic blood pressure, diastolic blood pressure, BMI, glycosylated hemoglobin, blood urea nitrogen, total cholesterol, serum calcium, total triglyceride, total protein, serum glucose, serum uric acid, and serum creatinine were adjusted.

To further investigate the non-linear relationships, an adjusted smooth curve plot model was exploited, and subgroup analyses were stratified by age, sex, and race. The association between UCR and the prevalence rate of kidney stones followed an inverted U-shaped curve in aged 40–59 years (inflection point: UCR 14.321 mmol/L) (Table 4 and Figure 5), women (inflection point: UCR 2.829 mmol/L) (Table 4 and Figure 2), and other race (inflection point: UCR 8.221 mmol/L) (Table 4 and Figure 3). This study demonstrated an inverted w-shaped curve after non-linear data fitting in the ungrouped model (inflection point: UCR 8.221 mmol/L) (Table 4 and Figure 1), men (inflection point: UCR 5.923 and

**TABLE 4:** Two-piecewise linear regression and logarithmic likelihood ratio test explained the threshold effect analysis of UCR on the renal stone incidence.

Urine Creatinine	ULR Test	PLR Test	LRT test
	OR (95% CI)	OR (95% CI)	*p*-value
<8.221 mmol/L	1.015 (1.008,1.021)	1.068 (1.042,1.095)	<0.0001
>8.221 mmol/L		1.003 (0.994,1.012)	
<22.896 mmol/L	1.003 (0.992,1.014)	0.994 (0.977,1.010)	0.167
>22.896 mmol/L		1.019 (0.994,1.043)	
20–39 years			
<5.658 mmol/L	1.011 (0.999,1.024)	1.217 (1.085,1.366)	<0.001
>5.658 mmol/L		0.999 (0.984,1.014)	
>21.923 mmol/L	1.019 (0.991,1.048)	0.929 (0.857,1.007)	0.016
<21.923 mmol/L		1.054 (1.017,1.092)	
40–59 years			
<14.321 mmol/L	1.018 (1.007,1.029)	1.044 (1.023,1.065)	0.003
>14.321 mmol/L		0.989 (0.966,1.012)	
White race			
<8.221 mmol/L	1.008 (1.001 1.014)	1.059 (1.033,1.086)	<0.001
>8.221 mmol/L		0.996 (0.987,1.005)	
<22.365 mmol/L	0.996 (0.983,1.009)	0.977 (0.955,0.999)	0.040
>22.365 mmol/L		1.018 (0.995,1.042)	
Other race			
<8.221 mmol/L	1.008 (1.001,1.014)	1.059 (1.033,1.086)	<0.001
>8.221 mmol/L		0.996 (0.987,1.005)	
Male			
<5.923 mmol/L	1.014 (1.005,1.023)	1.128 (1.049,1.213)	0.003
>5.923 mmol/L		1.007 (0.996,1.017)	
<33.062 mmol/L	1.031 (0.990, 1.075)	1.133 (1.053,1.218)	0.002
>33.062 mmol/L		0.867 (0.752,1.001)	
Female			
<2.829 mmol/L	1.015 (1.005, 1.025)	1.366 (1.083,1.723)	0.009
>2.829 mmol/L		1.011 (1.000, 1.021)	
<13.26 mmol/L	1.012 (1.001, 1.022)	1.026 (1.004,1.048)	0.131
>13.26 mmol/L		0.999 (0.980,1.019)	

*ULR, univariate linear regression; PLR, piecewise linear regression; LRT, logarithmic likelihood ratio test, statistically significant: p < 0.05.*

33.062 mmol/L) (Table 4 and Figure 2), aged 20–39 years (inflection point: UCR 5.658 and 21.923 mmol/L) (Table 4 and Figure 5), and white race (inflection point: UCR 8.221 and 22.365 mmol/L) (Table 4 and Figure 3). The association between UCR and the prevalence rate of kidney stones followed an inverted U-shaped curve with the inflection point of 106.08 μmol/L for serum creatinine (Table 5 and Figure 4).

**TABLE 5:** Two-piecewise linear regression and logarithmic likelihood ratio test explained the threshold effect analysis of serum creatinine on UCR.

Serum creatinine	ULR Test	PLR Test	LRT test
	β (95% CI)	β (95% CI)	*p*-value
<106.08 μmol/L	0.005 (0.002,0.008)	0.081 (0.074,0.088)	<0.0001
>106.08 μmol/L		−0.016 (−0.020, −0.013)	

*ULR, univariate linear regression; PLR, piecewise linear regression; LRT, logarithmic likelihood ratio test, statistically significant: p < 0.05.*

## Discussion

High incidence, high recurrence, and variable prognosis are the representative characteristics of nephrolithiasis (1, 14). Delay in treatment can lead to severe renal function deterioration. Early study has proposed kidney stones as a complex chronic systemic illness (15). Currently, the treatment options for renal calculi include surgery, physical, and drugs. Advancements in technology helped in the evolution of the operation approach from traditional open surgical methods to the various minimally invasive endoscopic procedures. However, the present treatment modality of kidney stones mainly targets clinical symptoms, and no etiological treatment is routinely available. The primary factor responsible for the formation and recurrence of kidney stones still remains unexplored (16). Therefore, elucidating the relevant risk factors of kidney stones reflects great clinical significance.

This program aimed to investigate the relationship between UCR and renal calculi. We excavated and analyzed large, organized, population-based cross-sectional data from the NHANES database. We observed a higher level of UCR in the kidney stone group relative to the non-stone group. The most significant finding of our study highlighted UCR as a risk factor for kidney stone formation, obtained by constructing multivariate logistic models. This association persisted even after adjusting for all of the confounders.

Subsequently, we performed a stratified analysis of the effects of UCR on kidney stones in several categories, and the findings revealed that the effects of UCR on kidney stones differed across groups. The impact of UCR on kidney stone development indicated a threshold effect between 40 and 60 years old (Figure 5), whereas there was a substantial linear association between >60 years old that may occur. As you may know, UCR typically grew and subsequently dropped with age (17), and UCR is positively connected with the occurrence of kidney stones, and thus, it is understandable why there is a threshold effect. Furthermore, age has been identified as a risk factor for kidney stone production. When a person reaches old age, UCR levels steadily drop. Diabetes and hypertension associated with aging are other established risk factors for kidney stone development (18). Age may have a considerably bigger influence on kidney stones than UCR at this time, which results in a straight increase trend in UCR and kidney stones. A threshold effect was found between UCR and kidney stone formation in both men and women, but the curves were different, which makes us think that sex hormones may have had an effect. Another NHANES study looked at testosterone levels in 10,193 people who took part. Hormone and the risk of kidney stone formation were linked. Multiple regression analysis found that there was no relationship between sex hormones (testosterone and estradiol) and the history of kidney stones in men or women (19). The result was against what this study found. One possible reason is that the variables and sample sizes in this study are not the same as the variables and sample sizes in this study. In order to be sure that a specific gender is linked to UCR and kidney stones, we need to do an RCT study. Finally, we discovered that although the incidence of UCR and kidney stones increased dramatically in whites, it decreased significantly in blacks, which indicates that the impact of various races on kidney stones is also significantly different. Our findings are comparable with those of prior studies (2). This might be because non-Hispanic blacks (men and women) have a lower urine excretion of calcium and oxalic acid than whites. There might be ethnic variations in gastrointestinal oxalate absorption, endogenous oxalate production, urinary citrate excretion, and relative susceptibility to parathyroid hormone effects (2).

Creatinine is a product of the muscle metabolic processes excreted by the kidney (20). It can be detected in the serum and urine. Under normal circumstances, a positive relationship can be derived between serum and urine concentrations. However, impaired kidney function may reverse this association. As reported by Ram B Jain, in chronic kidney disease stages 1–3, a significant positive association was noted between serum and UCR, while there was a change from positive to negative in stages 4–5 (10). An inverted U-shaped curve was obtained for the association between serum and UCR in our study. The result suggested that there was a threshold effect; the inflection point was serum creatinine 106.08 μmol/L. This was consistent with the previously reported result (10).

Persistent renal injury is one of the most serious and common complications of kidney stones. Chronic renal failure can result in the absence of timely, adequate treatment. UCR is the commonly employed endogenous marker to assess the severity of renal failure (21). With reliably detectable UCR, especially significantly higher levels in patients with kidney stone compared with the normal population, it is essential to determine the impact of UCR on kidney stone formation. Unfortunately, very limited evidence is available linking UCR with kidney stone formation. Available literature entirely relied on descriptive studies. In another database from NHANES, Mao et al. (5) documented increased UCR level in the kidney stone group, and univariate logistic regression analysis revealed a close relationship between UCR and the prevalence of kidney stones, but the authors did not establish a multivariate logistic regression analysis model to evaluate the relationship between the two. The relative mechanism is yet unknown. One of the main elements in stone formation is urine supersaturation (11). The rate of UCR excretion correlated positively with the rate of urine crystalloid excretion (calcium, magnesium, phosphate, uric acid) (22). Our research found greater UCR in stone groups than non-stone groups. Uric acid levels were also greater than the control group. This shows that urine ions in kidney stone formers may be supersaturated. If supersaturation conditions were maintained, it might crystallize in the renal pelvis. Clearly, further research is required to test this concept.

Univariate and multivariate logistic regression analyses in our study also confirmed some of the confounders to be predisposing factors for kidney stone formation. Among the demographic variables considered in this study, male, white race, married status, diabetes, and obesity were the strong risk factors for kidney stone mortality. These risk factors were in accordance with those described previously (23). Among the various clinical variables examined, in addition to the UCR, only total triglyceride was significantly correlated with renal stone occurrence. A significant association of high total cholesterol and triglycerides with a higher uric acid stone rate was reported from one study in the United States (24). However, the association between total cholesterol and kidney stone in our study was unremarkable. This study employed a questionnaire solely to detect stone formation and failed to involve the classification of renal calculi. This is one reason that might contribute to the differences noted between the two studies.

To our knowledge, this work describes the first attempt to investigate the influence of UCR in kidney stones occurrence. The large sample size also necessitated numerous subgroup analyses by ethnicity, age, and sex to search for a suitable group population. Furthermore, we explore potential non-linear relationships *via* smooth curve fittings and two-piecewise linear regression. This is the main advantage of our study. Nevertheless, some shortcomings also need to be pointed out. First, NHANES database is cross-sectional in design, and thereby, the causal relationship remains unclear. Second, the history of kidney stones in NHANES was just “self-reported” data, which excludes asymptomatic stones. Another limitation is that we failed to obtain the type of renal calculi. Third, eating habits could influence UCR; however, dietary information-associated variables were not acquired (25).

In conclusion, it is easy to carry out the urine routine test and is relatively inexpensive. It also emphasizes several parameters extremely useful in a clinical current routine examination. Significant elevation of UCR levels was evident in patients with nephrolithiasis. Kidney stone formation was associated with a high increase of UCR. Saturation and threshold effects in this relationship existed, and its effects also carried varied forms in different subgroups. Combined with subgroup analysis, white race, men, and older than 40 years adults imposed a higher risk of incidence.

## Data Availability Statement

Publicly available datasets were analyzed in this study. This data can be found here: www.cdc.gov/nchs/nhanes/.

## Ethics Statement

The studies involving human participants were reviewed and approved by the NCHS Ethics Review Board. The patients/participants provided their written informed consent to participate in this study.

## Author Contributions

XS, YanC, YZ, KX, and YangC performed the material preparation, collected the data, and analyzed the data. ZH wrote the first draft of the manuscript. All authors contributed to the study conception and design and commented on the previous versions of the manuscript, and read and approved the final manuscript.

## Conflict of Interest

The authors declare that the research was conducted in the absence of any commercial or financial relationships that could be construed as a potential conflict of interest.

## Publisher’s Note

All claims expressed in this article are solely those of the authors and do not necessarily represent those of their affiliated organizations, or those of the publisher, the editors and the reviewers. Any product that may be evaluated in this article, or claim that may be made by its manufacturer, is not guaranteed or endorsed by the publisher.

## Footnotes

1
www.cdc.gov/nchs/nhanes/

2
http://www.r-project.org/

3
http://www.empowerstats.com
